# Inflammatory ratios as predictors of length of hospitalization in psychiatric patients: A multicenter study

**DOI:** 10.1007/s00406-025-02033-9

**Published:** 2025-06-05

**Authors:** Ayse Kurtulmus, Fatma Busra Parlakkaya Yildiz, Rabia Kevser Sancili Boyraz, Zulal Celik, Aynur Gormez

**Affiliations:** 1 Department of Psychiatry, Istanbul Medeniyet University, Istanbul, Türkiye; 2 Department of Genetics, Aziz Sancar Enstitute of Experimental Medicine, Istanbul University, Istanbul, Türkiye; 3 Department of Psychiatry, Basaksehir Cam and Sakura City Hospital, Istanbul, Türkiye

**Keywords:** Psychiatric admission, Length of stay, Inflammation, NLR, PLR, MLR, Blood cells

## Abstract

**Introduction:**

Burgeoning evidence underscores the role of inflammation in psychiatric disorders where inflammation is linked to some clinical parameters such as disease severity and treatment resistance. However, the relationship between inflammation and the length of hospitalization remains poorly understood.

**Objectives:**

This study aimed to investigate whether elevated inflammation at the time of admission, as indicated by blood cell ratios which are easily accessible markers of peripheral inflammation, predicts the length of stay (LOS) in acutely ill psychiatric patients.

**Methods:**

This multi-centre study was conducted at two state hospitals using retrospective data. A total of 497 inpatients were screened, and clinical records at admission were carefully reviewed to exclude any intervening infections or inflammatory conditions. Patients were excluded if they had significantly elevated acute-phase reactants, symptoms of infection, received treatment for suspected infections, used anti-inflammatory drugs, or had physical examination findings suggestive of infection. Additionally, patients with alcohol/substance use disorders and those hospitalized after a suicide attempt were not included. Ultimately, the final sample comprised 167 individuals. CRP levels and NLR, MLR and PLR ratios derived from routine laboratory tests.

**Results:**

The length of hospitalization ranged from 10 to 85 days, with a mean of 30.43 ± 14.45 days. A significant positive correlation was found between LOS and NLR (r = 0.453, p <.001), PLR (r = 0.351, p <.001), and MLR (r = 0.292, p <.001), even after controlling for age, gender, diagnosis, comorbid medical conditions, and study site. However, CRP levels did not correlate with LOS (r = 0.025, p =.762). Hierarchical regression analysis revealed that adding the set of immune ratios significantly improved the model's predictive value (p <.001), with immune ratios explaining an additional 10.8% of the variance in LOS, even after controlling for other factors. No significant differences in NLR, PLR, or MLR were observed across diagnostic groups (p =.47, p =.52, and p =.15, respectively). No significant difference was observed between diagnostic groups in terms of inflammatory ratios.

**Conclusions:**

Our findings indicate that an elevated inflammatory response, as reflected by blood cell ratios, is associated with prolonged hospitalization in all patients, regardless of their diagnosis. These results emphasize the importance of addressing inflammatory processes in psychiatric care. Targeting inflammation as a modifiable risk factor may offer a new therapeutic avenue to reduce LOS. Future studies should focus on prospective designs and randomized controlled trials to determine whether targeting inflammation can directly reduce LOS and improve recovery in psychiatric populations.

**Supplementary Information:**

The online version contains supplementary material available at 10.1007/s00406-025-02033-9.

## Introduction

The average length of stay (LOS) for psychiatric admissions is roughly double that of other medical diagnoses [1]. Hospital admissions therefore are a significant contributor to mental health care costs, with approximately 29% of the £2.44 billion annual cost of severe mental disorders attributed to mental health inpatient bed days [2]. Beyond the economic burden on society, prolonged LOS can also negatively impact health outcomes. Many patients report that extended hospitalizations are unpleasant and stigmatizing [3]. Therefore, reducing LOS remains a key objective in mental health care. To address this, the National Health Services (NHS ) Mental Health Implementation Plan in the UK (2019) has set a target of reducing psychiatric hospital stays to a maximum of 32 days [4]. Identifying factors associated with extended hospitalizations may help in achieving this goal.

In recent years, numerous studies have emphasized the role of inflammation in psychiatric disorders. Abnormal inflammatory responses have been observed in conditions such as depression, bipolar disorder, and schizophrenia, classifying these disorders as chronic low-grade inflammatory diseases [5]. Evidence of altered inflammation-related markers across psychiatric conditions [6–8], along with reports of anti-inflammatory effects from psychotropic medications [9, 10] and the ameliorative impact of anti-inflammatory drugs on psychiatric symptoms [11–14], suggests a strong link between abnormal inflammatory responses and major psychiatric disorders. This abnormal inflammatory activity may represent a common intermediate pathway in the pathogenesis of these disorders. Large-scale genome-wide association studies have also identified associations between immune system-related genes and psychiatric conditions [15, 16]. Moreover, studies have shown that abnormal inflammation is associated with clinical parameters such as disease severity and treatment outcomes [17, 18]. Thus, inflammation appears to play a key role in the onset, progression, and treatment response in a variety of psychiatric disorders.

Given the evidence for abnormal inflammatory response in psychiatric disorders, the potential use of accessible markers to assess systemic immune status has gained increasing attention. Blood cell ratios, such as the neutrophil-to-lymphocyte ratio (NLR), monocyte-to-lymphocyte ratio (MLR), and platelet-to-lymphocyte ratio (PLR), derived from routine hemograms, have been utilized as indicators of peripheral inflammation [19–21]. The numbers and proportions of circulating blood cells—such as neutrophils, thrombocytes, and lymphocytes—may vary in response to inflammation. These ratios are valuable because they are easily accessible and cost-effective compared to more complex and expensive measures, such as cytokine levels, which require specialized assays. Studies have shown that these ratios are reliable markers of inflammatory status, correlating well with other established inflammatory markers like C-reactive protein (CRP) and cytokine levels [22, 23]. Recent research has also reported significant differences in NLR, MLR and PLR ratios between patients with psychiatric disorders and healthy controls [19, 21, 24]. While these ratios have been linked to clinical course and prognosis in conditions such as cardiovascular disease, cancer, and autoimmune disorders, their association with the clinical course and prognosis in psychiatric disorders remains unclear, with available data yielding conflicting results [25–28].

The aim of this study was to examine the relationship between blood cell ratios and the length of hospitalization in acutely ill psychiatric patients. To the best of our knowledge, this is the first study to specifically investigate the association between a set of blood cell ratios-such as NLR, MLR and PLR- and duration of hospitalization in this patient population. Our primary hypothesis is that an elevated inflammatory response, as indicated by these ratios, will be associated with prolonged length of hospitalization, independent of the diagnosis. To test this, we focused on a highly selective cohort, excluding comorbidities and other factors that could influence inflammatory markers. Establishing this association may be critical for identifying potential interventions that could help reduce length of hospitalization in psychiatric care.

## Materials and methods

### Participants

This multicenter study, which aimed to investigate the relation of inflammatory parameters with the length of hospitalization, independent of disease diagnosis, was conducted at two different state hospitals in Istanbul. Ethical approval was granted by Istanbul Medeniyet University Goztepe Research and Training Hospital with the number of 2023/0872. Data were retrospectively collected from inpatients admitted to the psychiatric department of Goztepe Research and Training Hospital between January 2022 and June 2023 and from inpatients admitted to the psychiatric department of Basaksehir Cam and Sakura Hospital between February and December 2023. Goztepe Research and Training Hospital has an inpatient unit for only females, whereas Başakşehir Çam and Sakura Hospital accomodates both female and male wards.

A priori power analyses revealed that a total sample of 133 patients were required (a = 0.05, power = 0.95, effect size = 0.10 with 8 predictors).

All patients hospitalized during the specified period were screened for eligibility. The study included patients aged 18 to 65, the majority of whom were diagnosed with ICD-10 psychotic disorders (F20-29) or affective disorders (F30-39). All diagnosis were made by a consultant psychiatrist based on ICD-10 criteria. Patients were excluded if they were discharged due to treatment refusal or readmitted within two weeks of discharge. Only the first admission was included for patients with multiple hospitalizations during the study period. Additionally, patients with a diagnosis of alcohol or substance use disorder, or those hospitalized following a suicide attempt, were excluded due to the potential impact of these conditions on inflammatory parameters. Other exclusion criteria included the presence of chronic inflammatory diseases, acute or chronic infectious diseases, elevated acute phase reactants, treatment with corticosteroids or immunosuppressants, receipt of blood transfusions during hospitalization, and pregnancy.

**Fig. 1:**
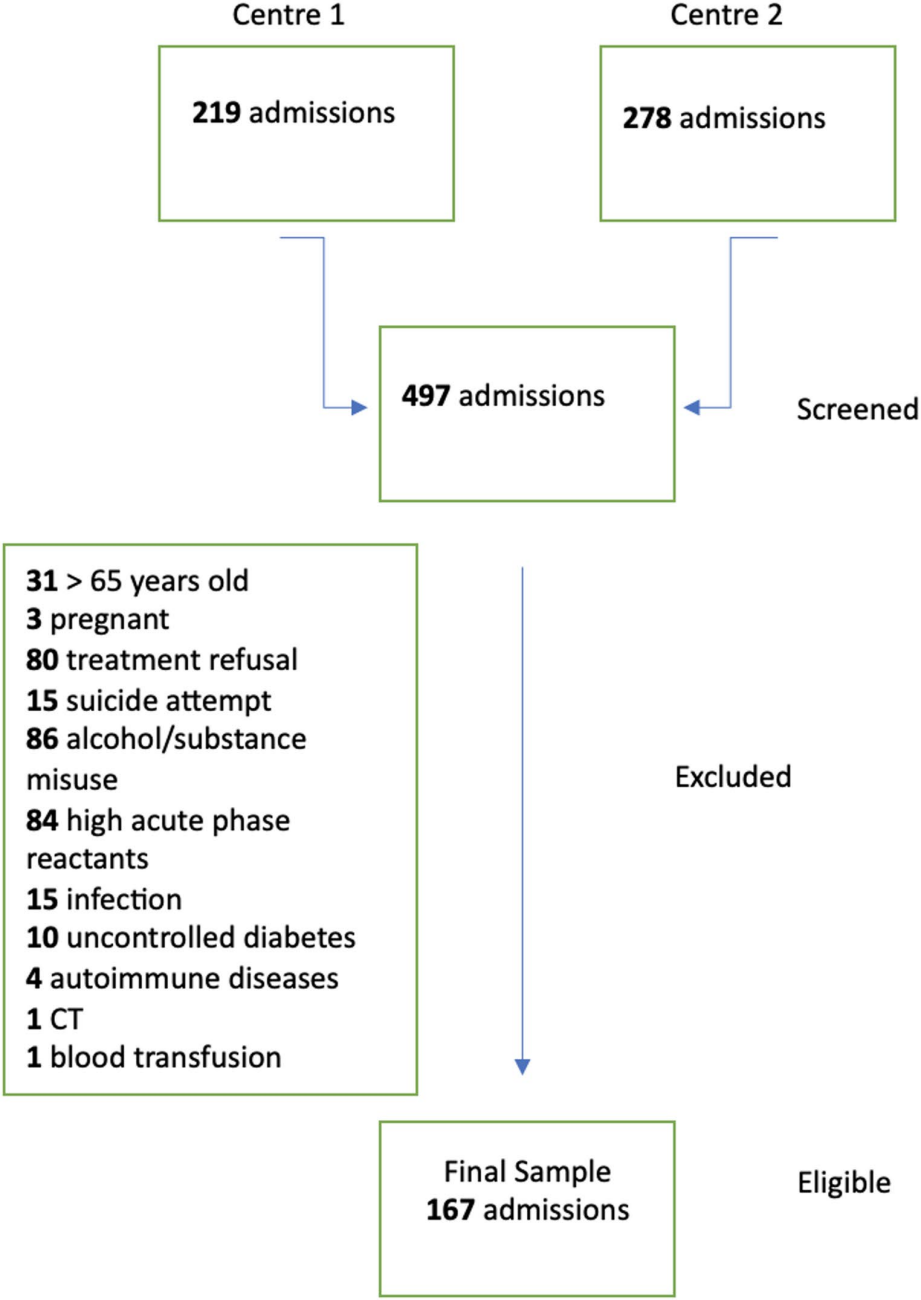
Flow chart of study sample

A total of 497 patients from the two centres were screened. After considering for study eligibility, 330 individuals were excluded from the study, and the final sample consisted of 167 individuals (Fig. 1).

### Procedures

Sociodemographic data, psychiatric diagnoses, comorbid conditions, and length of hospital stay were obtained retrospectively from patients’ clinical records. Due to the study’s retrospective design, a comprehensive review of clinical records at the time of admission—including discharge summaries, consultation notes, laboratory assessments, daily clinical progress, and nursing observation notes—was conducted to exclude any cases of intervening infections or inflammations. Patients were excluded if they had significantly elevated acute phase reactants, such as CRP (> 7 mg/L), erythrocyte sedimentation rate (> 20 mm/hr), or leukocytosis (> 12000 cells/µL) in laboratory tests; exhibited symptoms of infection during their clinical course; were consulted or treated for a suspected infection; used anti-inflammatory medications; had positive physical examination findings suggestive of infection; or presented with fever.

CRP levels and complete blood counts from routine tests performed during hospitalization were evaluated retrospectively. Only blood samples collected within the first 24 h of hospitalization were included, with most samples drawn between 8:00 and 9:00 a.m. The blood cell ratios used in the study were calculated as follows: Neutrophil-to-Lymphocyte Ratio (NLR) = neutrophils/lymphocytes (absolute values), Platelet-to-Lymphocyte Ratio (PLR) = platelets/lymphocytes (absolute values), and Monocyte-to-Lymphocyte Ratio (MLR) = monocytes/lymphocytes (absolute values).

### Statistical analyses

All analyses were performed using the IBM SPSS for Macintosh, version 25.0. Descriptive characteristics of the study sample were reported as means ± standard deviations (SD) for continuous variables and percentages for categorical variables. The normality of the variable distributions was assessed using the Kolmogorov-Smirnov test. Differences between diagnostic groups were examined using either ANOVA or the Kruskal-Wallis test, depending on the distribution of the dependent variables. Pearson’s correlation coefficient was employed to evaluate the relationship between inflammatory parameters and length of hospitalization. In order to account for potential confounders, multiple linear regression analyses were performed to control for age, sex, diagnosis, comorbid medical conditions, and study site.

Hierarchical regression analysis was conducted to assess the proportion of variance in the length of hospitalization explained by the immune ratios (NLR, MLR, and PLR), after adjusting for age, sex, diagnosis, comorbidities, and study site. Additionally, receiver operating characteristic (ROC) curve analysis was utilized to evaluate the predictive value of the immune ratios for prolonged hospitalization. A median split was applied to define prolonged hospitalization, and the area under the ROC curves (AUC) was used to assess the predictive value of these ratios.

## Results

### Descriptives

A total of 497 patients were screened for eligibility, of which 167 met the inclusion criteria and were analyzed (Fig. 1). The length of hospitalization ranged from 10 to 85 days, with a mean of 30.43 ± 14.45 days. The primary diagnoses among the patients were as follows: 45.5% had psychotic disorders (38 schizophrenia, 7 schizoaffective disorder, 2 delusional disorder, 26 non-organic psychotic disorder, 1 acute and transient psychotic disorder), 28.7% had bipolar disorders, 19.8% had depressive disorders, and 6% had other diagnoses, which included 2 cases of eating disorders, 1 case of obsessive-compulsive disorder, 3 cases of anxiety disorders, 3 cases of other mood disorders, and 1 case of adjustment disorder.

Given that one of the study sites had only a female inpatient ward, 79.6% of the final sample consisted of female patients. The mean age of the participants was 37.60 ± 11.89 years, and only 12% had a comorbid medical condition.

In the overall sample, 13 patients had subsequent admissions within the study period. Only the first admission of these patients was included in the primary analyses to avoid data duplication. However, we conducted a post hoc analysis to investigate whether patients with multiple admissions during the study period differed from others in terms of their inflammatory parameters. Our findings indicated no significant differences in inflammatory parameters between patients with subsequent hospitalizations and those without in any of the inflammatory parameters *(p =.60*,* 0.76 and 0.97 for NLR*,* MLR and PLR*,* respectively).*

### Length of hospitalization by diagnostic groups

The mean length of hospitalization by diagnosis was as follows: 33.89 ± 15.49 days for psychotic disorders, 27.87 ± 9.81 days for bipolar disorder, 26.21 ± 13.88 days for depressive disorders, and 30.20 ± 20.21 days for other diagnoses. A significant difference in length of hospitalization was observed across diagnostic groups (H = 8.92, *p* =.03). Post hoc analyses revealed that patients with psychotic disorders had a longer hospitalization compared to those with bipolar and depressive disorders. However, only the difference between psychotic disorders and depressive disorder was statistically significant (Z = -1.86, *p* =.009), while the difference between psychotic disorder and bipolar disorder remained at a trend level (Z = -2.60, *p* =.062).

### Association between length of hospitalization and inflammatory parameters

There was a significant positive correlation between the length of hospitalization and NLR (*r* =.453, *p* <.001), PLR (*r* =.351, *p* <.001), and MLR (*r* =.292, *p* <.001). However, CRP levels were not significantly correlated with the length of hospitalization (*r* =.025, *p* =.762). Multiple linear regression analyses confirmed that the associations between each blood cell ratio and length of hospitalization remained significant after adjusting for age, sex, diagnosis, comorbid medical conditions, and study site (Table 1). Partial regression plots of each immune ratios on the length of hospitalization has been provided in Supplementary Fig. 1.

**Table 1:** Association between inflammatory parameters and length of hospitalization

	Univariate analyses		Multivariate analyses^1^				
	r	*p*	Unstandardized B	Standardized B	t	*p*	CI
***NLR***	0.45	***< 0.001***	3.90	0.29	4.02	***< 0.001***	1.98–5.81
***MLR***	0.29	***< 0.001***	42.18	0.29	4.11	***< 0.001***	21.90-62.45
***PLR***	0.35	***< 0.001***	0.06	0.22	2.82	***0.005***	0.02-0.10
***CRP***	0.03	0.76					

^1^after controlling for age, sex, diagnosis, comorbid medical diseases, study site.

### Predictors of length of hospitalization

A hierarchical regression analysis was conducted to determine how much variance in the length of hospitalization could be explained by a set of inflammatory ratios (NLR, MLR, and PLR) after controlling for age, sex, diagnosis, comorbid medical conditions, and study site. The first model included age, sex, diagnosis, comorbid medical diseases and study site and the second model was established by adding NLR, MLR and PLR into the first model. The analysis revealed that the inclusion of inflammatory ratios significantly improved the predictive value of the model (*p* <.001), with immune ratios explaining an additional 10.8% of the variance in length of hospitalization, even after controlling for the effects of other variables. In terms of the unique contribution of variables to the equation MLR has emerged as the only ratio that has a statistically significant predictive value when the overlapping effects of all other variables removed (*p* =.014) (Table 2).

**Table 2:** Hierarchical regression analyses on the predictors of length of hospitalization

	Unstandardized B	Standardized B	t	*p*	CI
**Model 1** ^**a**^					
(Constant)	46.77		8.09	< 0.001	35.36, 58.19
Age	− 0.15	− 0.12	-1.65	0.101	− 0.33, 0.03
Sex	-1.52	− 0.04	− 0.43	0.670	-8.54, 5.51
Diagnosis (Ref: psychotic disorder)					
Bipolar disorder	-6.14	− 0.19	-2.45	**0.016**	-11.10,-1.18
Depressive disorder	-8.73	− 0.24	-3.03	**0.003**	-14.42,-3.04
Others	-4.06	− 0.07	− 0.89	0.375	-13.07, 4.95
Comorbid medical disease	11.39	0.26	3.50	**< 0.001**	4.97, 17.82
Study site	-4.80	− 0.16	-1.60	0.112	-10.73, 1.14
**Model 2** ^**b**^					
(Constant)	30.72		4.56	< 0.001	17.40, 44.03
Age	− 0.12	− 0.10	-1.33	0.186	− 0.29,0.06
Sex	− 0.42	− 0.01	− 0.12	0.904	-7.29, 6.45
Diagnosis (Ref: psychotic disorder)					
Bipolar disorder	-4.88	− 0.15	-2.02	**0.045**	-9.65,-0.11
Depressive disorder	-8.73	− 0.24	-3.18	**0.002**	-14.16,-3.30
Others	-5.16	− 0.09	-1.18	0.240	-13.79,3.47
Comorbid medical disease	10.19	0.23	3.26	**0.001**	4.01,16.37
Study site	-5.15	− 0.17	-1.77	0.079	-10.89,0.60
NLR	2.08	0.15	1.46	0.146	− 0.73, 4.89
MLR	29.26	0.20	2.49	**0.014**	6.04,52.47
PLR	0.01	0.05	0.51	0.613	− 0.04,0.07

^a^ R^2^:0.168, SE:13.38, R^2^ Change:0.168, *p* <.001

^b^ R^2^:0.275, SE:12.61, R^2^ Change:0.108, *p* <.001

### Predictive value of inflammatory ratios for length of hospitalization

**Fig. 2:**
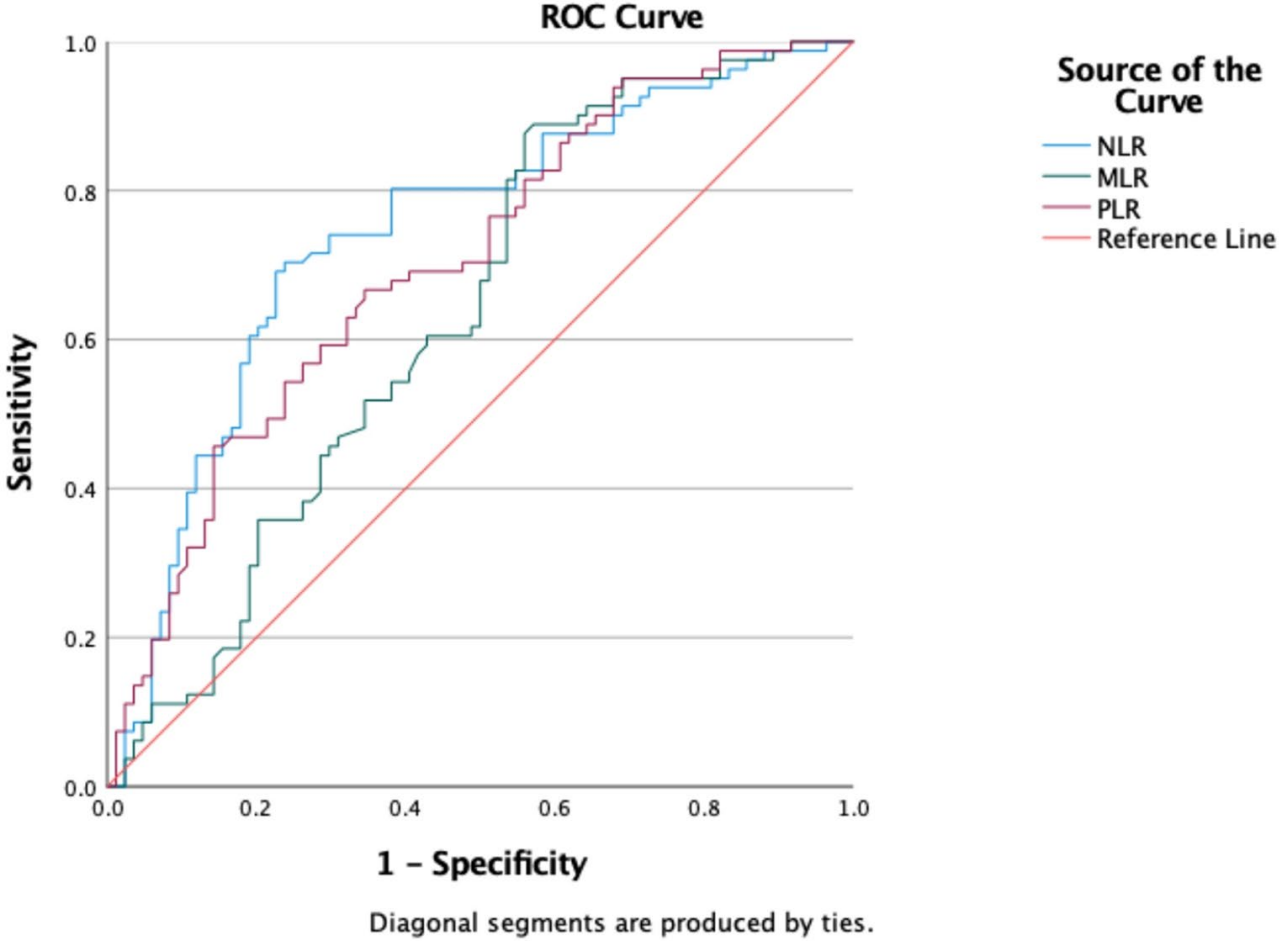
ROC curves for immune ratios in predicting prolonged hospitalization

Patients were categorized into two groups based on their length of hospitalization, with those exceeding the median length of stay classified as having a prolonged hospitalization. Figure 2 presents the area under the ROC curves (AUC) for immune ratios in predicting prolonged hospitalization. Both NLR and PLR demonstrated an AUC greater than 0.7, indicating good predictive accuracy. The ROC characteristics were as follows: NLR AUC = 0.74 (95% CI: 0.67–0.82, *p* <.001) and MLR AUC = 0.70 (95% CI: 0.63–0.78, *p* <.001).

### Inflammatory ratios by diagnostic groups

With regards to group differences in immune cell ratios, no significant difference was observed between diagnostic groups in either NLR, MLR or PLR (*p* =.47, *p* =.52 and *p* =.15, respectively) (Supplementary Fig. 2).

## Discussion

Our findings indicate that the inflammatory status of patients at the time of admission may serve as a predictor for the length of hospitalization in psychiatric inpatients. Elevated levels of markers such as the NLR, MLR, and PLR, which are routinely obtained through standard clinical assessments and are both easily accessible and widely utilized, were associated with prolonged hospital stays. To the best of our knowledge, this is the first study to demonstrate a relationship between a set of inflammatory cell ratios and the duration of hospitalization in acute psychiatric patients.

The growing body of literature has led to increased interest in the blood cell ratios as indicators of inflammation. Meta-analyses consistently demonstrate that these ratios are elevated in diverse psychiatric patient groups, such as bipolar disorder, schizophrenia and major depression, compared to healthy controls [19–21, 24]. However, the relationship between inflammatory blood cell ratios and clinical parameters, such as severity and stage of illness, remains unclear. Studies has indicated that NLR levels are elevated in patients with both first-episode psychosis and chronic schizophrenia, with this elevation remaining consistently high throughout the course of the illness; however, a slight decrease in NLR levels is observed following remission and with long-term antipsychotic treatment [24]. Similar trends have been observed for the MLR and PLR. Studies have demonstrated that both MLR and PLR increase during acute exacerbations and remain elevated after remission when compared to healthy controls, with levels being significantly higher during the exacerbation phase than in the remission phase for the same patients [29]. In a large population-based study, the leukocyte-to-lymphocyte ratio (LLR), NLR, and MLR ratios were found to be associated with mortality and treatment non-response in schizophrenia spectrum disorders [30]. However, data regarding the relationship between immune ratios and the stage and severity of illness in patients with depression appear to be more inconsistent. While some studies have reported no significant difference in NLR or PLR levels between outpatients and inpatients with depression, others indicate that elevated NLR is specific to first-episode depression, is not observed in recurrent episodes, and decreases with treatment [31, 32, 33]. Additionally, studies have produced mixed findings concerning the correlation between severity of depression and immune ratios [34–35]. In patients with bipolar disorder, the data regarding the relationship between these immune parameters and disease stage is also inconsistent. Some studies have reported elevated inflammatory blood cell ratios compared to controls in different stages of bipolar disorder, while a meta-analysis indicated that higher NLR and PLR values were predominantly observed in studies that specifically included manic patients or those that considered all patients regardless of disease stage. In contrast, no significant differences between patients and controls were found in studies that focused solely on euthymic patients [21]. In inpatient settings, comparisons between patients admitted with acute mania and those admitted with depression revealed that NLR, MLR and PLR levels were higher in the former group [37]. These findings suggest that the increase in inflammatory ratios in patients with bipolar disorder is particularly pronounced during manic episodes. However, Melo et al. (2019) reported that NLR and PLR levels in euthymic bipolar patients correlated with anxiety levels, functioning, and the number of previous episodes and hospitalizations [38].

Although several studies have evaluated the association of inflammation with various clinical parameters—such as illness severity, duration, antipsychotic dosage, number of episodes, and hospitalizations—data specifically linking high inflammatory levels to length of hospitalization in psychiatric disorders remain limited. Adachi et al. (2018) reported that among elderly patients admitted to acute psychiatric wards, baseline elevations of C-reactive protein (CRP) were less prevalent in those who were discharged early [39]. The relationship between inflammatory ratios and length of hospitalization has been examined in other medical fields, revealing a correlation between these markers and duration of hospitalization across various patient populations, including those with acute ischemic stroke, myocarditis, appendicitis, diabetic foot ulcers, and post-cardiac surgery [40–44]. However, to the best of our knowledge, this is the first study to investigate the relationship between length of hospitalization and a set of immune ratios in psychiatric patients, independent of diagnosis. Our findings indicate that a heightened inflammatory response is transdiagnostically associated with prolonged hospitalization across all patient groups.

In our study, we found no significant differences in inflammatory ratios between the diagnostic groups. This finding seems to align with existing literature. A large cross-sectional study demonstrated elevated NLR levels across various diagnostic groups compared to controls; however, no differences were evident between the diagnostic subgroups [44]. Bulut et al. (2021) compared NLR and PLR levels in bipolar mania, bipolar depression, and schizophrenia, reporting that these ratios were elevated in all patient groups relative to healthy controls. Nonetheless, they observed no differences in PLR between the diagnostic groups, while NLR was significantly higher in the bipolar mania group compared to the others [45]. These findings support the notion that inflammation may serve as a common intermediary pathway, reflecting a transdiagnostic pathological process in psychiatric disorders, although the extent of involvement may vary across different conditions. Therefore, the role of inflammation should be carefully considered in the management of psychiatric patients. Even if inflammation is not a primary factor in the pathophysiology of these disorders and is merely elevated as a result of an acute relapse, the heightened inflammatory response appears to exert a detrimental sustaining effect on psychopathology. Consequently, intervening to address inflammation during the acute phase may be associated with a more rapid response and improved outcomes by disrupting this vicious cycle and mitigating the additional negative effects of the inflammatory burden on patients. Indeed, studies examining anti-inflammatory therapies in psychiatric disorders show promising results. For instance, the addition of anti-inflammatory agents in patients with schizophrenia has been linked to improvements in general, negative, and cognitive symptoms [46]. Similarly, comparable or even more pronounced benefits have been reported in bipolar disorder and depression with adjunctive anti-inflammatory treatments. Data from these studies suggest that adjunctive anti-inflammatory drugs may facilitate favorable treatment responses, particularly in subgroups of patients exhibiting high levels of inflammation [47]. However, an important question remains regarding whether these agents merely expedite treatment responses or contribute to more substantial long-term improvements. The former hypothesis is supported by studies demonstrating that while no significant differences in efficacy were observed between treatment groups at the end of the study, notable differences in response were evident during the initial weeks of anti-inflammatory treatment [48, 49]. In any case, these findings indicate that incorporating anti-inflammatory agents into the treatment of acute psychiatric illnesses may not only provide a faster rate of improvement but also positively influence economic, social and psychological dimensions by modifying disease-related outcomes, including the length of hospitalization.

Our post hoc analyses revealed no significant difference in inflammatory ratios between patients between multiple versus single admissions during the study period. However, caution should be exercised when interpreting these results. Firstly, the number of patients who had subsequent hospitalisation was low (*n* = 13), which may have resulted in statistical tests being underpowered. Secondly, the data only includes patients who were readmitted to the same hospital, and it is not known whether any other patients were readmitted to different psychiatric wards. Moreover, as the inflammatory ratios are known to fluctuate over time and are not regarded as stable traits, immune cell ratios at the time of first admission may not serve as a reliable indicator for subsequent admissions and long-term course of the disease.

We acknowledge several limitations in our study. The primary limitation is its retrospective design, which may have restricted our ability to fully account for the effects of concomitant acute infections and other confounding factors. To address this, we conducted a thorough evaluation of clinical records, including mental health assessments, clinical review notes, clinical tests, and laboratory investigations, while applying strict exclusion criteria. Furthermore, the retrospective nature of the study allowed for the blinding of the treatment team, which helped reduce potential investigator bias related to the length of hospitalization. Another limitation is that we did not assess disease severity and treatment response as separate variables. While disease severity could mediate the relationship between inflammation and length of hospitalization, our study focused primarily on establishing this association rather than identifying specific mediators. Future studies may benefit from exploring the relationship between disease severity, immune ratios, and the correlation between changes in immune ratios and treatment response. Additionally, we did not assess BMI and smoking status in our patient population—both of which can influence inflammatory parameters and are often skewed in psychiatric populations compared to the general population. Although increased BMI and smoking are well-established factors affecting leukocyte counts, the primary objective of our study was to investigate whether increased inflammatory ratios at admission could predict the length of hospitalization, regardless of the underlying causes of this abnormal inflammatory status. Future research incorporating relevant covariates, such as BMI and smoking status, would provide a more comprehensive understanding of these associations. Lastly, it is worth noting that one of the participating centers only had a female ward, resulting in a predominantly female study population. However, regression analyses indicated that the length of hospitalization predicted immune ratios independently of sex. Despite this, our findings should be validated in male patient populations with larger sample sizes.

## Conclusions

In conclusion, our findings indicate that elevated levels of inflammation are associated with the need for prolonged hospitalization in acute psychiatric patients, irrespective of diagnosis. Therefore, interventions aimed at targeting inflammation in these patients may alleviate both the economic and psychosocial burdens associated with extended hospitalization by reducing length of hospitalization. However, the results of this pioneering study require further validation through additional research.

## Electronic supplementary material

Below is the link to the electronic supplementary material.

Supplementary Material 1
