# Supplementary material for: Inflammatory ratios as predictors of length of hospitalization in psychiatric patients: A multicenter study
Source: Eur Arch Psychiatry Clin Neurosci. 2025 Jun 5;276(1):77–86. doi: 10.1007/s00406-025-02033-9 (PMC12904961; doi:10.1007/s00406-025-02033-9)
Supplement: Supplementary file 1 — Supplementary Material 1 [file 406_2025_2033_MOESM1_ESM.docx]

**a.**


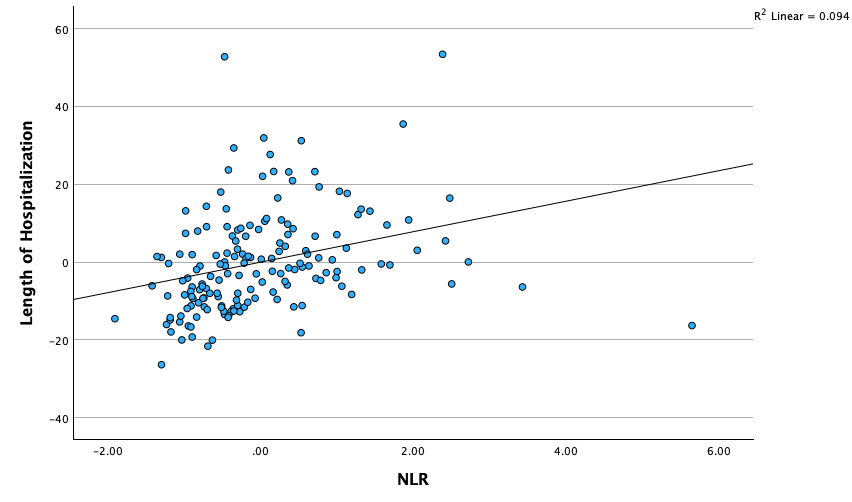


**b.**


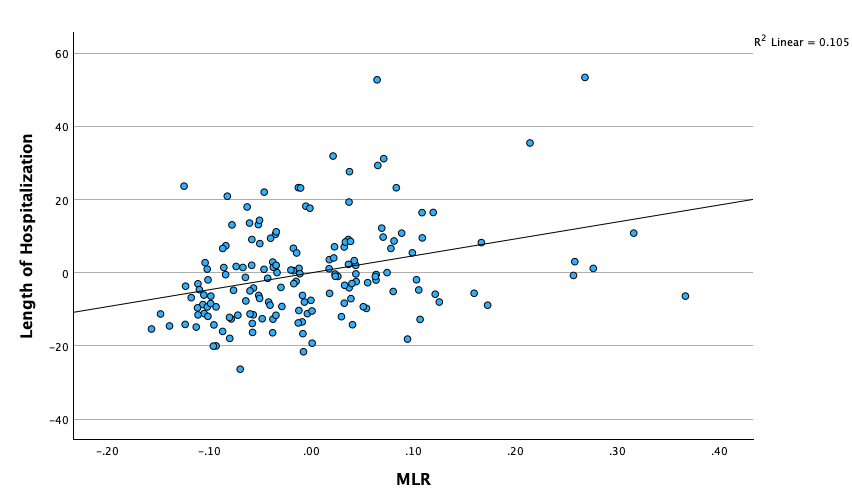


**c.**


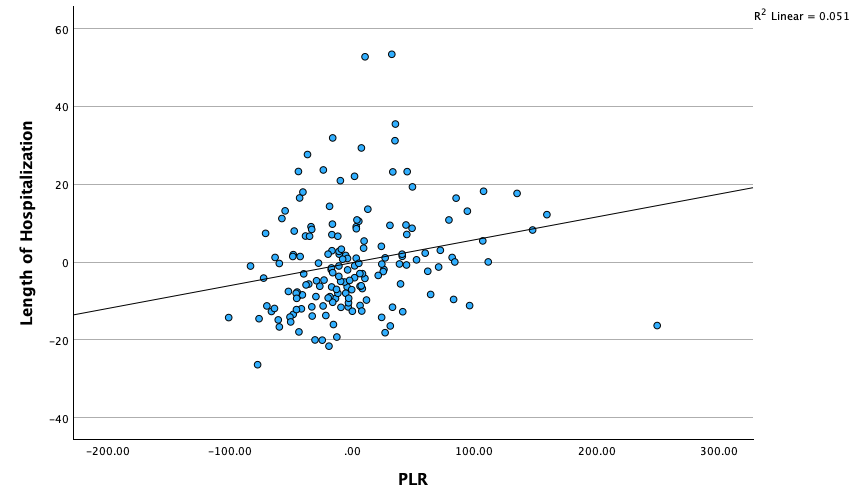


***Supplementary Figure-1.*** Partial regression plots indicating the relationship between immune ratios and length of hospitalization after controlling for age, sex, diagnosis, comorbid medical diseases and study site. **a**. NLR, **b**. MLR and **c**. PLR


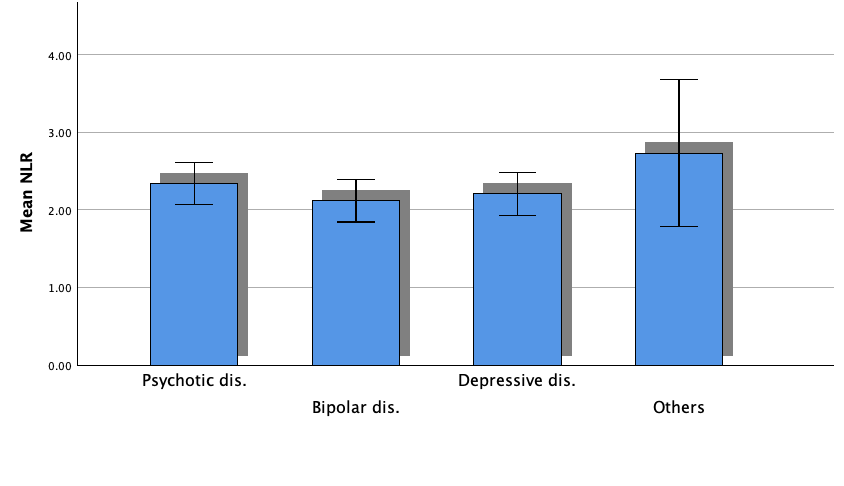


**a.**

***Supplementary Figure-2***. Comparison of NLR (**a**), MLR (**b**) and PLR (**c**) between diagnostic groups.


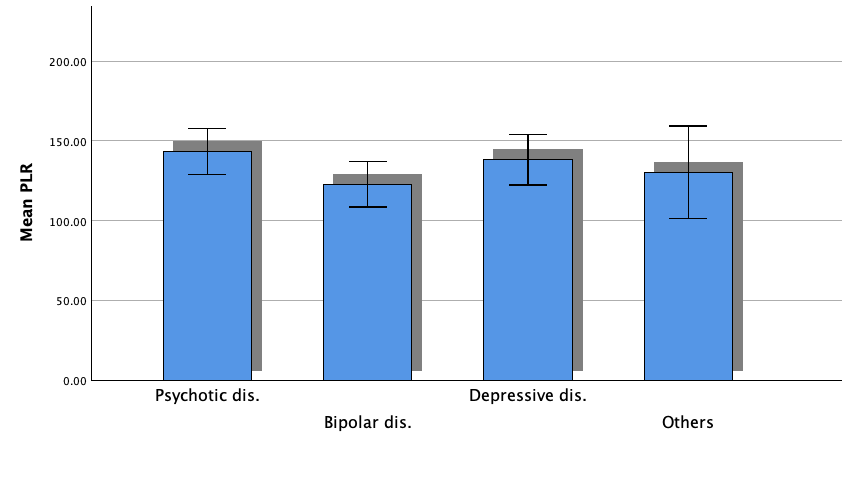

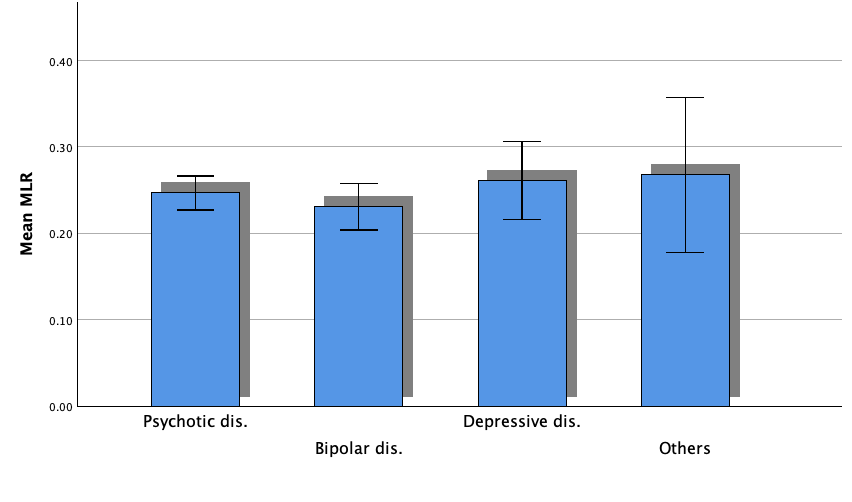


**c.**

**b.**
